# SARS-CoV-2 Infection and Guillain-Barré Syndrome: A Review on Potential Pathogenic Mechanisms

**DOI:** 10.3389/fimmu.2021.674922

**Published:** 2021-05-10

**Authors:** Shahrzad Shoraka, Maria Lucia Brito Ferreira, Seyed Reza Mohebbi, Amir Ghaemi

**Affiliations:** ^1^ Basic and Molecular Epidemiology of Gastrointestinal Disorders Research Center, Research Institute for Gastroenterology and Liver Diseases, Shahid Beheshti University of Medical Sciences, Tehran, Iran; ^2^ Department of Microbiology and Microbial Biotechnology, Faculty of Life Sciences and Biotechnology, Shahid Beheshti University, Tehran, Iran; ^3^ Department of Neurology, Hospital da Restauração, Recife, Brazil; ^4^ Gastroenterology and Liver Diseases Research Center, Research Institute for Gastroenterology and Liver Diseases, Shahid Beheshti University of Medical Sciences, Tehran, Iran; ^5^ Department of Virology, Pasteur Institute of Iran, Tehran, Iran

**Keywords:** Coronavirus, COVID-19, SARS-CoV-2, Neurological manifestations, Guillain-Barré syndrome

## Abstract

Since December 2019, the world has been facing an outbreak of a new disease called coronavirus disease 2019 (COVID-19). The COVID-19 pandemic is caused by a novel beta-coronavirus named severe acute respiratory syndrome coronavirus 2 (SARS-CoV-2). The SARS-CoV-2 infection mainly affects the respiratory system. Recently, there have been some reports of extra-respiratory symptoms such as neurological manifestations in COVID-19. According to the increasing reports of Guillain-Barré syndrome following COVID-19, we mainly focused on SARS-CoV-2 infection and Guillain-Barré syndrome in this review. We tried to explain the possibility of a relationship between SARS-CoV-2 infection and Guillain-Barré syndrome and potential pathogenic mechanisms based on current and past knowledge.

## Introduction

Over the past two decades, coronaviruses have caused three epidemic diseases named the severe acute respiratory syndrome (SARS), the Middle East respiratory syndrome (MERS), and coronavirus disease 2019 (COVID-19) (1). All these three diseases are caused by coronaviruses belonging to the beta genus (2, 3). Infections caused by these beta-coronaviruses show a variable range of clinical manifestations, from being asymptomatic to severe disease and death (4). Although pulmonary symptoms are considered the main clinical manifestation, neurological complications associated with these three respiratory coronaviruses have also been reported (2, 5).

In this review, we mainly focused on SARS-CoV-2 infection and Guillain-Barré syndrome and tried to explain the potential pathogenic mechanisms based on current and past knowledge.

## Virology of SARS-CoV-2

The coronavirus family consists of enveloped viruses with a positive single-stranded large RNA genome (6–8). Coronaviruses cause a wide variety of diseases in humans and some animals (9). Some of them have highly host-specific, while others are found on a range of hosts (10). According to genetic and serological properties, these viruses are divided into four subfamilies: α, β, γ, and δ, while the beta genus is also divided into four lineages of A, B, C, and D. Human coronavirus (HCoV) infections are caused only by alpha and beta genera (11, 12). Evidence indicated that the beta genus have more severe symptoms and complications compared to other genus (2). Besides, HCoVs are classified as zoonotic pathogens (13). Although these viruses cause respiratory infections in humans, their ability to affect other host organs such as heart, liver, gastrointestinal tract, kidney, Central and Peripheral Nervous System makes them complex pathogens (2, 5, 14–17).

In December 2019, the emerging virus causing COVID-19 was added to the coronavirus family. The virus, called SARS-CoV-2 belongs to the beta group, same as SARS-CoV and MERS-CoV, but to the lineage B (3). Analyses showed the SARS-CoV-2 is about 80% similar to the SARS-CoV. Both of these viruses enter the host cell by binding its surface spike protein to the host angiotensin converting enzyme-2 receptor (ACE-2). However, the binding affinity of the SARS-CoV-2 spike protein to the ACE-2 receptor is higher than that seen in SARS-CoV. Also it has recently been shown that SARS-CoV-2 may utilize basigin (BSG; CD147) and neuropilin-1 (NRP1) as binding receptors (18). Compared to SARS-CoV and MERS-CoV, the SARS-CoV-2 has higher transmissibility and pathogenicity (2, 3, 19–22).

During the current pandemic, most patients with COVID-19 show respiratory symptoms such as dry cough and shortness of breath. SARS-CoV-2 infection has clinical manifestations similar to those reported for SARS and MERS. Therefore, these three viruses are mainly known as respiratory pathogens. However, they could contribute to symptoms and complications related to other organs, especially in severe cases (2, 5, 23, 24). Gastrointestinal, cardiac, hepatic, kidney, ocular, cutaneous, and haematological, symptoms are the main extra-respiratory manifestations of patients with COVID-19 (8, 25–27). Recently, neurological symptoms were reported in some COVID-19 cases, raising concerns about the potential of the SARS-CoV-2 to invade nerves and lead to neurological complications, both in the acute and chronic phases. The term “neuro-COVID” is used to describe these complications (28, 29).

## Neurovirulence of Human Coronaviruses

The prevalence of neuro-COVID has been reported to vary between studies. Although the prevalence rate of neurological symptoms is estimated to be around 3.5 to 84% among COVID-19 patients, in most cases the SARS-CoV-2 RNA was not detected in the cerebrospinal fluid (CSF) (28, 30, 31). Among 58 patients with COVID-19 and neurological symptoms, the SARS-CoV-2 RNA was detected in CSF of 2 patients (3.4%). One patient with refractory headache and another with ADEM four days after the onset of COVID-19 symptoms (32). Also Domingues et al. detected SARS-CoV-2 in CSF using by RT-PCR and confirmed with deep sequenced. There was a 99.74 to 100% similarity between the patient virus to the worldwide sequences (33). On the other hand, organoids and *in vivo* studies in human ACE2 transgenic mice have demonstrated that the SARS-CoV-2 could infect neurons and contribute to cell death and neural damage. However, CSF and autopsy findings do not provide consistent support for direct CNS invasion. So SARS-CoV-2 related neurological symptoms may be the consequences of different mechanisms (18, 29, 34).

Given that coronaviruses could lead to short- or long-term neurological disorders, there was a hypothesis that these viruses may have neurovirulence because of the neurotropism and neuroinvasion of human coronaviruses (35–39). Since 2000, Arbour et al. found HCoV RNA in brain samples outside blood vessels, and affirmed the consistence of neuroinvasion by these respiratory pathogens in humans but considered the need of further studies to distinguish between opportunistic and disease-associated viral presence (40).

**Figure 1:**
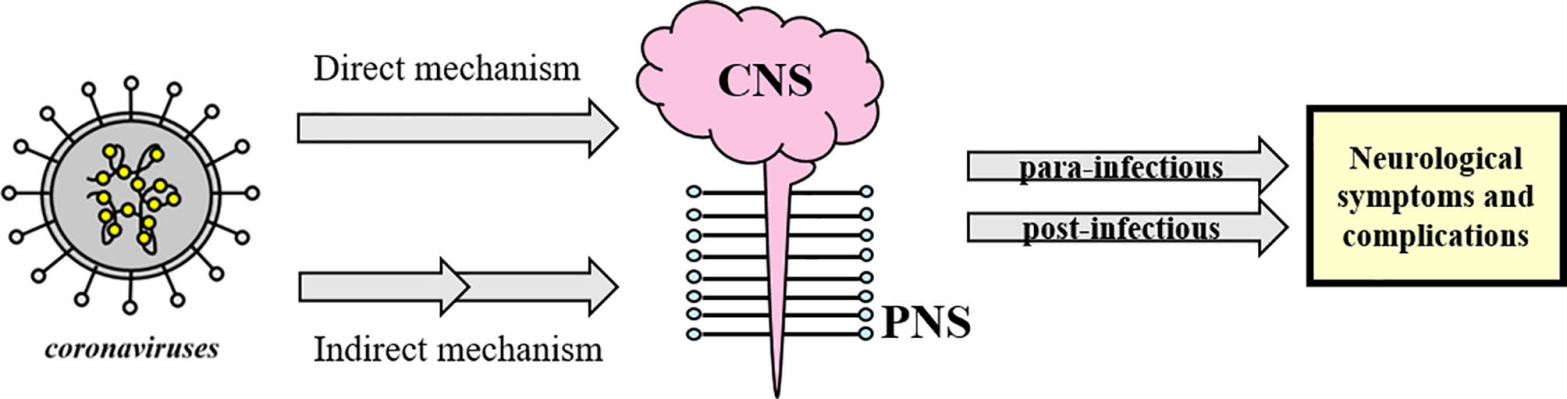
Schematic classification of neurological complications associated with coronavirus infections. Coronaviruses could directly or indirectly affect the CNS and/or PNS and lead to post- or para-infectious neurological complications. The concept appears to be generalizable to SARS-CoV-2 as well.

It seems that coronaviruses could be responsible for direct and indirect neurological symptoms and complications with central nervous system (CNS) (including headache, epileptic seizure, impaired consciousness and dizziness) and peripheral nervous system (PNS) manifestations (such as Guillain-Barré syndrome, anosmia and neuralgia) (41), divided as para-infectious and post-infectious (5, 8, 23, 34, 42–45) (see **Figure 1**).

During the SARS-CoV and MERS-CoV outbreak, some neurological symptoms were among their other extra-pulmonary complications (**Table 1**). At the present SARS-Cov-2 outbreak, there have been increasing reports of clinical neurological disorders such as acute encephalomyelitis, acute flaccid paralysis (AFP), multiple sclerosis (MS), acute demyelinating encephalomyelitis (ADEM), and Guillain-Barré syndrome (GBS) as possible complications, supporting its neuroinvasion nature (12, 40, 51–53). Among them, Guillain-Barré Syndrome is the most frequent.

**Table 1:** Neurological symptoms attributable to SARS-CoV-2 published in 2020 and 2021.

Authors	Attributable neurological symptoms
**Mao et al. (** 46 **)**	Neurological manifestations involving CNS and PNS present in 78 of 214 (36.4%) patients. The neurological symptoms were more common (45.5%) in those with more severe infection
**Karadas et al. (** 47 **)**	Cerebrovascular disorders, epileptic seizures, impaired consciousness; dizziness, olfactory and taste disorders were neurological symptoms. These symptoms were present in 83 of the 239 (34.7%) patients and headache was the most common (27.6%)
**Rifino et al. (** 48 **)**	Cerebrovascular disease, peripheral nervous system disease, altered mental status, and miscellaneous disorders present in 137 of 1,760 COVID-19 patients
**Chen et al. (** 49 **)**	Consciousness disorders were present on admission in 22% of patients who died compared with 1% who recovered
**Chuang et al. (** 50 **)**	Out of 282 patients with neurological problems, 56 had a COVID-19 positive test. Of these, 23 patients had no symptoms of COVID-19 while 33 had. In both groups, weakness of consciousness was the most common primary neurological symptom

## Guillain-Barré Syndrome and SARS-CoV-2 Infection

Guillain-Barré syndrome is an acute acquired autoimmune disorder of the peripheral nerves that often occurs after infection (54). In fact, GBS is symmetrical ascending paralysis, often caused by respiratory or gastrointestinal infections from a virus or bacteria (4). Many bacteria and viruses have been considered as possible trigger of GBS (55, 56). Before the recent pandemic, few cases of coronavirus associated to GBS were reported, but a systematic review pointed a significant increasing number of patients with GBS after the COVID-19 pandemic, with higher prevalence among older patients (mean age of 60 years) than with younger ones (mean age of 40 years) (57) (**Table 2**).

**Table 2:** Cases of GBS associated to coronavirus.

Authors	Year	Attributable neurological symptoms
**Before the recent pandemic**		
**Kim et al. (** 58 **)**	2017	After a severe infection, 55-year-old man with a positive test for MERS-CoV showed hypersomnolence, ophthalmoplegia and relative symmetric motor weakness in all four limbs. Given the infection history the GBS variant was considered a possible diagnosis. The patient had progressive symmetric external ophthalmoplegia, ataxia, and impaired consciousness with limb weakness, and the diagnosis of Bickerstaff’s encephalitis (BBE) overlap with GBS was suggested.
**Sharma et al. (** 53 **)**	2019	A 5-year-old boy with a 7-day history of bilateral lower limb pain, irritability, difficulty walking, and loss of balance, 4 days after left facial droop and inability to close the left eye as well as fever and a stuffy nose two weeks earlier. Diagnosis of GBS was supported by areflexia and albuminocytologic dissociation. The positivity of multiplex PCR for HCoV-OC43 indicated that coronavirus infection may have caused atypical GBS
**During the recent pandemic, GBS post a SARS-CoV-2**		
**Coen et al. (** 59 **)**	2020	A 70-year-old man suffered from myalgia, fatigue, and dry cough and 10 days after he was hospitalized for paraparesis, distal allodynia, voiding problem, and constipation. Since his SARS-CoV-2 test was positive before the first signs of polyneuropathy, it supports a post-infectious GBS phenotype
**Camdessanche et al. (** 60 **)**	2020	A 64-year-old man had fever and cough after a lesion in his shoulder and evolved with moderate dyspnea. His nasopharyngeal swab was positive for SARS-CoV-2. After five days without fever, he complained of paresthesia in feet and hands and within three days he had a flaccid severe tetraparesia. Five days after the electrodiagnosis of GBS was confirmed.
**Sedaghat and Karimi (** 61 **)**	2020	A 65-year-old man had a positive SARS-CoV2 test. Twelve days after he presented to the emergency room symptoms of acute progressive symmetric ascending quadriparesis. Neurological and laboratory findings supported the diagnosis of GBS
**Padroni et al. (** 62 **)**	2020	A 70-year-old woman had fever and cough and a positive result of the nasopharyngeal swab for SARS-CoV-2. After few weeks she presented GBS symptoms. Microbiological tests on her CSF were negative
**Rifino et al. (** 48 **)**	2020	17 out of 31 COVID-19 patients with peripheral neuropathies, had GBS
**Khalifa et al. (** 63 **)**	2020	An 11-year-old boy had a diagnosis of GBS associated with SARS-CoV-2 infection
**Alberti et al. (** 64 **)**	2020	A 71-year-old man had a mild fever for a few days. One week after he was referred to the emergency department with paresthesia at the limb and flaccid tetraparesis. Nasopharyngeal swab was positive, but CSF was negative for SARS-CoV-2
**Toscano et al. (** 65 **)**	2020	Of five patients, who developed GBS after the onset of COVID-19 symptoms, four had a positive nasopharyngeal swab for SARS-CoV-2 at the time of neurologic symptoms and a negative CSF. The interval between the onset of viral infection and the first symptoms of GBS was five to ten days
**Filosto et al. (** 66 **)**	2020	Of 34 patients with GBS diagnosed during the outbreak of SARS-CoV-2 in 12 referral hospitals in seven cities in northern Italy, 30 patients (88.2%) were diagnosed with confirmed SARS-CoV-2. The incidence of GBS in March and April 2020 in northern Italy has increased 2.6 fold compared to same months of 2019
**Dufour et al. (** 67 **)**	2021	A 36-year-old woman with progressive ascending weakness consistent with GBS. She had recovered from mild COVID-19 two weeks ago
**Tekin et al. (** 68 **)**	2021	A 34-year-old woman who developed COVID-19 at 37th gestational week was diagnosed with GBS at postpartum
**Raahimi et al. (** 69 **)**	2021	A 46-year-old man was diagnosed with GBS 53 days after having COVID-19. This is a case report of delayed onset of GBS following the SARS-CoV-2 infection
**During the recent pandemic, GBS para-infectious paralysis**		
**Zhao et al. (** 70 **)**	2020	A 61-year-old woman with acute weakness in both legs and a diagnosis of GBS. On the eighth day, the patient started having a dry cough and fever. Then, the oropharyngeal swab test for SARS-CoV-2 was positive.
**Ottaviani et al. (** 71 **)**	2020	A 66-year-old woman who presented GBS neurological symptoms one week after the onset of respiratory symptoms while they progressed simultaneously
**Bastug et al. (** 72 **)**	2021	A 66-year-old man with GBS neurological findings occurred on the third day of the COVID-19 diagnosis

**Figure 2:**
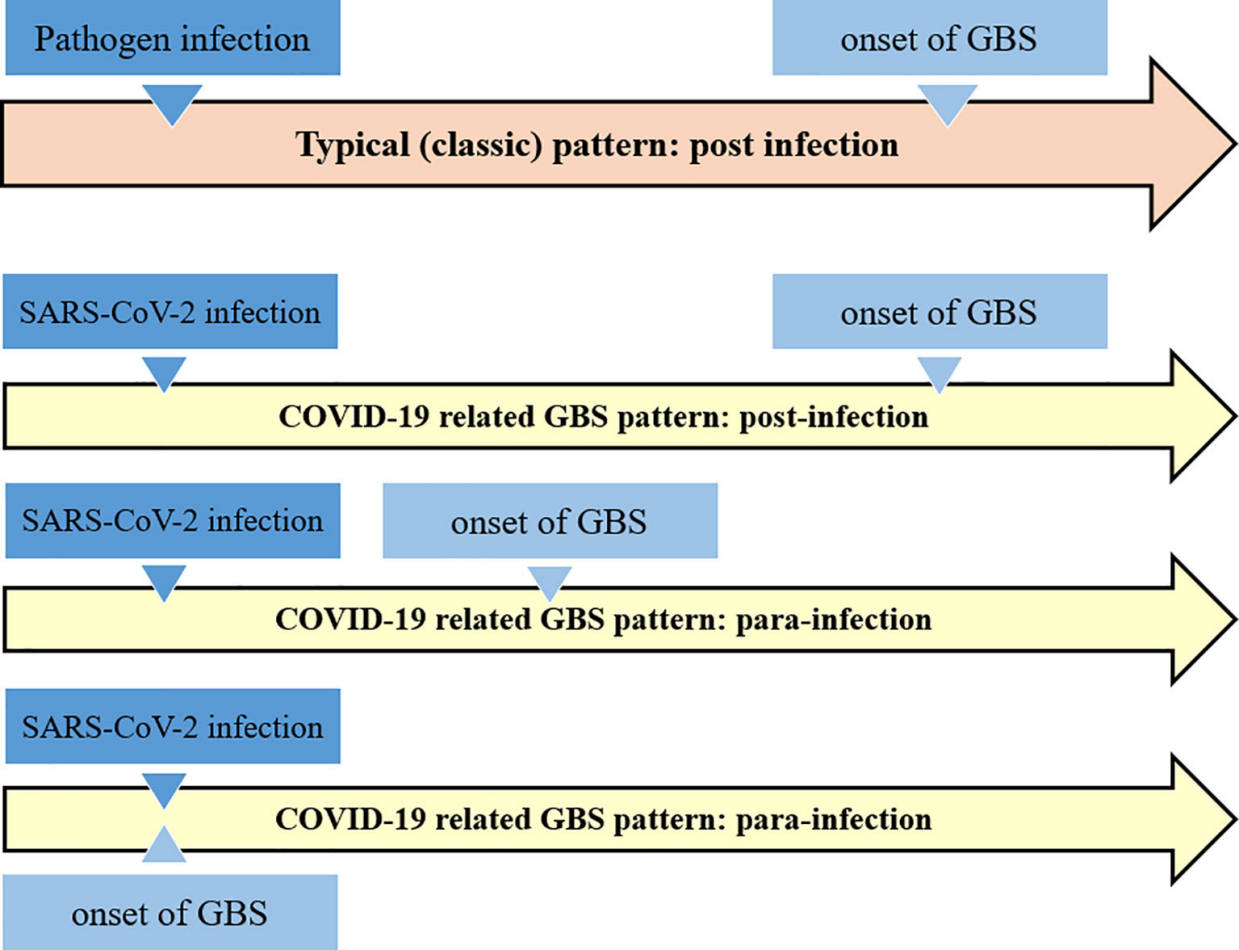
Schematic comparison of the classical Guillain-Barré syndrome pattern with the COVID-19 related GBS. It appears that COVID-19 related GBS follow the both post- and para-infections patterns.

GBS associated to SARS-CoV-2 infection may follow the typical post-infectious pattern, with report indicating that it is also possible in children. GBS has also been reported as part of the “long COVID-19 syndrome” (69, 73). Nevertheless, there are also reports that GBS is a para-infectious paralysis associated with a viral infection (74). Almost all reported cases have acute onset within a few days of onset of viral infection (75) (see **Figure 2**).

### Possible Mechanisms of Guillain-Barré Syndrome Involving Virus and Bacteria

Molecular mimicry between microbial and neural antigens is a major driving force in this disorder. The interaction between microbial agents and the host that dictates the immune response to the unwanted auto reactivity is not well understood yet. On the other hand, most people (>99%) who are exposed to immune stimulus, as a result of GBS-associated infection do not develop unwanted autoimmunity. It seems that genetics and environmental factors affect the susceptibility of individual to this disease (56, 76, 77).

The association of various microorganisms with Guillain-Barré syndrome has been reported such as *Campylobacter jejuni (C. jejuni)*, *Mycoplasma pneumoniae*, *Haemophilus influenza*, Cytomegalovirus (CMV), Epstein-Barr virus (EBV), influenza A, varicella-zoster, hepatitis (A, B and E), Zika and Chikungunya viruses (78–91). *C. jejuni* has been identified as the most common pathogen causing this disorder (91, 92).

In 2001, Yuki showed that the core lipo-oligosaccharides of the *Campylobacter jejuni* strains associated with GBS have structural similarities to various gangliosides in peripheral nerve membranes, suggesting that molecular mimicry of gangliosides may contribute to GBS (93). Molecular mimicry of gangliosides in *C. jejuni* results in the production of anti-ganglioside antibodies that bind to gangliosides in the axonal membrane at the Ranvier node. Activation of complement leads to disruption of voltage-gated sodium channels, disruption of nodal structure, and formation of the membrane attack complex that leads to calcium influx. Eventually, these changes cause axonal damage and attract macrophages, which could then migrate between axon and myelin (80, 94). Guillain-Barré-associated viral infections show similar mechanisms to bacterial GBS. However, due to the wide range of viral antigens that may be associated with GBS, the pathophysiology, the clinical course, and outcomes may vary (78).

Studies have described the role of GM2 anti-glycoside antibodies in the pathogenesis of CMV-related GBS. The findings of these studies showed that anti-GM2 IgM antibodies are induced in acute cytomegalovirus infection through molecular mimicry between GM2 and the antigens induced by CMV infection (77, 93, 95, 96). For hepatitis A, B, C, or E, a definite homogeneous epitope with components of the peripheral nerve has not yet been described. However, in 2009, Loly et al. reported the presence of anti-ganglioside GM2 antibodies in hepatitis E virus–related GBS. They have declare a possible molecular mimicry of gangliosides (97).

Over the past decades, studies have demonstrated a possible link between the arthropod-borne viruses (arboviruses) including Zika and Chikungunya virus and the development of GBS (98–102). Shortly after the outbreak of ZIKV, in 2015, a cluster of cases with GBS was identified in Brazil (103). Also, a case-control study in France revealed that out of 42 patients diagnosed with Guillain-Barré syndrome, 41 (98%) had anti-Zika virus IgM or IgG, and all (100%) had neutralizing antibody against the Zika virus (104). The analysis of case series from seven countries showed that the changes in the reported incidence of Zika virus disease during 2015 and early 2016 were closely related to the change in the incidence of Guillain-Barré syndrome (105).

In the case of GBS associated with the Zika virus, several anti-ganglioside antibodies also are indicated and may play a role in the mechanism of molecular mimicry (104, 106, 107). In addition, there are other alternative mechanisms described for Zika-related GBS. It could be due to a direct viral neuropathic effect, and cross-reactive antibodies formed during previous infections with a detrimental effect on nerve function. The underlying mechanisms of para-infectious pathogenesis have not yet been identified. It has been suggested that specific Zika virus-peptides may not only cross-react but also induce a cellular immune response *via* antigen-presenting cell activation of T-lymphocytes (104, 106, 108, 109).

During the recent pandemic, there is also growing evidence that SARS-CoV-2 infection is associated with immune-mediated neurological complications, for example, in the form of GBS (65, 110). The exact pathogenesis of COVID-19-related neurological damage is still largely unknown. Considering that previous viral outbreaks, molecular mimicry between SARS-CoV-2 and various human organs and tissues have been hypothesized as a potential trigger of multi-organ autoimmunity in COVID-19 (104, 111–114). Moreover, failure to detect the SARS-CoV-2 in most of the CSF patient samples supports an immune mechanism rather than direct invasion (18).

In a recent study by Lucchese and Flöel, sequence analysis of the 41 human proteins associated with acute and chronic immune-mediated neuropathies revealed that SARS-CoV-2 contained two immunologically-related hexapeptides (KDKKKK in nucleocapsid and EIPKEE in Orf1ab) with the human heat shock proteins 90 (HSP90B and HSP90B2) and 60 (HSP60), respectively (113). These authors hypothesized that SARS-CoV-2 infection may trigger an adaptive immune response in which T cell-B cell interactions result in the production specific antibodies similar to ganglioside-peptide sequences or structure, resulting in loss of self-tolerance (113). The gangliosides located on the membranes of neurons and the Schwann-cells, which form the myelin sheath, act as receptors for antiganglioside antibodies, promoting neutralization of neurons complement inhibitory activity, which turn them into targets for autoimmune-mediated destruction of myelin sheaths or axons (113).

About 50 to 85% of previously reported cases with GBS or its variants have anti-ganglioside antibodies in their serum. However, there are limited data on the presence of anti-ganglioside antibodies in the patients with COVID-19 related GBS. Studies have not reported increase in the serum titers of anti-ganglioside antibodies in GBS patients with COVID-19. Recently, Dufour et al. reported first case with COVID-19 related GBS with positive GM1 antibody (67, 75). Therefore, further studies are necessary to confirm the presence of anti-ganglioside antibodies in COVID-19 related GBS.

This molecular mimicry has also been shown in the implication of HSPs in some immune-mediated clinical conditions (115). A 2015 study by Loshaj-Shala et al. reported a high homology between *C. jejuni* DnaK and GroEL with the human peripheral nerve HSP70 and HSP60, respectively. These results strongly support the potential role of chaperone molecules in the progression of the autoimmune response related to GBS (92). Also an etiological relationship between some neurological diseases and autoantibodies against HSP family proteins has sometimes been described (116, 117). It is noteworthy that autoantibodies targeting different families of HSPs were increased in serum and CSF of patients with GBS compared to healthy controls (118, 119). In a study, Yonekura et al. demonstrated that IgG and IgM antibody titers against several HSPs (including HSP60) were significantly higher in the CSF of patients with GBS in the acute phase than patients with motor neuron diseases (MND) (120). The sharing of peptide motifs with immunologic potential between SARS-CoV-2 and HSPs strongly supports immune-mediated neuronal damage (121).

The role of neuroinflammation and the effect of cytokine storm caused by SARS-CoV-2 infection on the nervous system have been discussed. In COVID-19 patients, an increase has been observed in cytokines such as interleukin-1β (IL-1β), IL-6, IL-17, TNF- α and interferon-γ (IFN-γ), along with other chemokines. Because many of the same cytokines have been implicated in the pathogenesis of typical GBS, the cytokine storm in COVID-19 may play a pivotal role in the simultaneous development and progression of GBS (75). Zhang et al. in 2013 reported elevated serum TNF-α levels during acute phase of GBS and it was associated with clinical severity of the disease (122). CSF levels of IFN-γ, IL-4, IL-17, and IL-22 are increased in GBS, and IL-17 and IL-22 levels are associated with disease severity (123–125). However, the role of cytokines in COVID-19 related GBS needs further investigation (126, 127).

Here, we describe the possible mechanisms that may be involved in the development of Guillain-Barré syndrome following the SARS-CoV-2 infection. Further studies are needed to better understand the possible mechanism of this relation. To confirm the exact pathogenic mechanism, identification of T cell responses, or particular antibodies to the target autoantigen; recognition of structural homology between the infectious agent and the target autoantigen and, finally, reproduction of the disease following immunization with the infectious agent in an animal model should be considered (128).

## Conclusion, Future Directions, and New Insights

During the recent pandemic, many cases of Guillain-Barré syndrome have been reported to be associated with COVID-19. In this review article, we have discussed the possibility of a relationship between SARS-CoV-2 infection and Guillain-Barré syndrome and potential pathogenic mechanisms based on current and past knowledge. It is noteworthy that during the recent pandemic, there have been several case reports of the para-infectious pattern of GBS according to SARS-CoV-2 infection. On the other hand, GBS may be part of the “long COVID-19 syndrome”. Therefore, Guillain-Barré syndrome associated with SARS-CoV-2 in addition to the classic post-infectious profile might follow the pattern of a para-infectious as reported in Guillain-Barré syndrome associated with the Zika virus. Identifying and linking para- and post-infectious neurological diseases such as GBS to a pandemic is confusing. Besides, accurate and timely diagnosis is critical. Therefore, considering that there may be a link between SARS-CoV-2 infection and GBS is helpful in rapid diagnosis of patients. We have also highlighted that the possible role of indirect immune- mediated mechanisms, such as molecular mimicry and neuroinflammation, is more than direct viral invasion in the development of COVID-19 related GBS. Also further molecular studies are needs to investigate the exact mechanism that leads to the GBS following the SARS-CoV-2 infection.

In conclusion, although many studies support the link between SARS-CoV-2 infection and GBS, more evidence is needed to confirm their relationship and describe its exact mechanism. Epidemiological evidence associated with the suspected infectious agent and GBS should be considered to approve the association between COVID-19 and GBS. However, based on recent reports, we suggest that all newly diagnosed Guillain-Barré cases should be tested for SARS-CoV-2 infection in the current pandemic, even if they have no respiratory complaints.

## Author Contributions

SRM: article decision, planning, and writing. SHSH: literature search and reviewing and article writing. MLBF: manuscript revision on Neurology. AGH: article reviewing. All authors contributed to the article and approved the submitted version.

## Conflict of Interest

The authors declare that the research was conducted in the absence of any commercial or financial relationships that could be construed as a potential conflict of interest.

## Abbreviations

COVID-19, coronavirus disease 2019; SARS-CoV-2, severe acute respiratory syndrome coronavirus 2, SARS, severe acute respiratory syndrome; MERS, Middle East respiratory syndrome; RNA, ribonucleic acid; HCoV, Human coronavirus; ACE-2, angiotensin converting enzyme-2 receptor; BSG, basigin; NRP1, neuropilin-1; CNS, central nervous system; PNS, peripheral nervous system; AFP, acute flaccid paralysis; MS, multiple sclerosis; ADEM, acute demyelinating encephalomyelitis; GBS, Guillain-Barré syndrome; RT-PCR, reverse transcriptase PCR; PCR, polymerase chain reaction; CSF, cerebrospinal fluid; BBE, Bickerstaff’s encephalitis; CoVs, coronaviruses; IL, interleukin; TNF-α, tumor necrosis factor-alpha; CMV, cytomegalovirus; EBV, Epstein-Barr virus; arboviruses, arthropod-borne viruses; ZIKV, zika virus; HSP, heat shock proteins; IFN-γ, interferon-γ; MND, motor neuron diseases.
